# Leukocyte Populations in Human Preterm and Term Breast Milk Identified by Multicolour Flow Cytometry

**DOI:** 10.1371/journal.pone.0135580

**Published:** 2015-08-19

**Authors:** Stephanie Trend, Emma de Jong, Megan L. Lloyd, Chooi Heen Kok, Peter Richmond, Dorota A. Doherty, Karen Simmer, Foteini Kakulas, Tobias Strunk, Andrew Currie

**Affiliations:** 1 Centre for Neonatal Research and Education, The University of Western Australia, Perth, Western Australia, Australia; 2 School of Paediatrics and Child Health, The University of Western Australia, Perth, Western Australia, Australia; 3 School of Veterinary and Life Sciences, Murdoch University, Perth, Western Australia, Australia; 4 School of Pathology and Laboratory Medicine, The University of Western Australia, Perth, Western Australia, Australia; 5 Neonatal Clinical Care Unit, King Edward Memorial Hospital for Women, Perth, Western Australia, Australia; 6 School of Women’s and Infants’ Health, The University of Western Australia, Perth, Western Australia, Australia; 7 School of Chemistry and Biochemistry, The University of Western Australia, Perth, Australia; Ludwig-Maximilians-Universität, GERMANY

## Abstract

**Background:**

Extremely preterm infants are highly susceptible to bacterial infections but breast milk provides some protection. It is unknown if leukocyte numbers and subsets in milk differ between term and preterm breast milk. This study serially characterised leukocyte populations in breast milk of mothers of preterm and term infants using multicolour flow cytometry methods for extended differential leukocyte counts in blood.

**Methods:**

Sixty mothers of extremely preterm (<28 weeks gestational age), very preterm (28–31 wk), and moderately preterm (32–36 wk), as well as term (37–41 wk) infants were recruited. Colostrum (d2–5), transitional (d8–12) and mature milk (d26–30) samples were collected, cells isolated, and leukocyte subsets analysed using flow cytometry.

**Results:**

The major CD45+ leukocyte populations circulating in blood were also detectable in breast milk but at different frequencies. Progression of lactation was associated with decreasing CD45+ leukocyte concentration, as well as increases in the relative frequencies of neutrophils and immature granulocytes, and decreases in the relative frequencies of eosinophils, myeloid and B cell precursors, and CD16- monocytes. No differences were observed between preterm and term breast milk in leukocyte concentration, though minor differences between preterm groups in some leukocyte frequencies were observed.

**Conclusions:**

Flow cytometry is a useful tool to identify and quantify leukocyte subsets in breast milk. The stage of lactation is associated with major changes in milk leukocyte composition in this population. Fresh preterm breast milk is not deficient in leukocytes, but shorter gestation may be associated with minor differences in leukocyte subset frequencies in preterm compared to term breast milk.

## Introduction

Feeding of human breast milk (BM) is associated with fewer infections and reduced gastrointestinal inflammation in preterm infants [1, 2]. Though all newborns have weak cellular and humoral defences, preterm infants are particularly susceptible to bacterial infections, with known deficiencies in adaptive and innate immunity including decreased leukocyte pool, lack of third trimester maternal immunoglobulin transfer, and decreased leukocyte cytokine and antibody production in response to bacteria [3–7]. BM contains a myriad of immunological, biochemical and cellular contents, which have the potential to significantly alter newborn immunity and susceptibility to infection. BM-derived leukocytes engulf and kill bacteria [8] and produce antimicrobial proteins and peptides (AMPs) [9]. In animal models, BM leukocytes can translocate from the gastrointestinal tract to blood and distant sites including the liver and spleen [10, 11]. Microscopy-based identification of BM leukocytes comparing very preterm (<32 wk gestational age; GA), moderately preterm (32–34 wk GA), and term colostrum suggests an inverse correlation between GA and the concentration of leukocytes [12]. However, it is not known how preterm birth affects the leukocyte populations in transitional or mature milk, or in milk after extremely preterm birth (<28 wk GA).

Several flow cytometry-based methods for differentiation of blood and bone marrow leukocytes have been recently described, that allow for the reliable, automated and extended differentiation of known leukocyte subsets [13–17]. Importantly, these methods have been validated against the gold standard of diagnostic cytology and allow for a robust measurement of an extended differential leukocyte count (up to 11 leukocytes subsets) in both healthy and diseased individuals [18]. These methods may allow for identification of a greater range of leukocytes in BM, especially in the presence of mammary gland epithelial cells and stem cells and achieve greater certainty and superior differentiation. Given the potential for maternal leukocytes to contribute to infant immunity, an extended characterisation of preterm BM leukocytes may contribute to our understanding of the susceptibility of preterm infants to infections.

The aims of this study were to assess the effectiveness of the use of an extended validated blood leukocyte differential staining panel, described by Faucher *et al*. [13], to identify the same leukocyte populations in human milk; to use this method to compare the total milk leukocyte concentration and subset frequencies in colostrum, transitional and mature milk, and; to investigate the effect of preterm birth and infection on these cell populations in milk.

## Materials and Methods

### Sample collection

The Women and Newborn Health Service Human Research Ethics Committee approved this study. Written informed consent was obtained from sixty women giving birth between 22–42 wk gestation before commencing any study procedures or sample collection. Inclusion criteria included maternal age ≥18 years, intention to breastfeed and absence of primary immune deficiencies, diabetes mellitus or known congenital abnormalities. Sixty participants were recruited across four groups based on WHO preterm birth categories [19]; extremely preterm (EP, <28 wk GA; n = 15), very preterm (VP, 28–31 wk GA; n = 15), moderately preterm (MP, 32–36 wk GA; n = 15) or term (37–41 wk GA; n = 15). Individuals with a history of other common conditions, particularly those associated with preterm birth such as infections and hypertension were not excluded, nor individuals taking medications, in order to gain a representative sample of the preterm population.

BM was collected from participants at three time points, selected to represent colostrum (C; day 2–5, ≤3.5 mL), transitional milk (TM; d8–12; ≤7 mL) and mature milk (MM; d26–30; ≤7 mL), based on the categories described by Castellote *et al*. [20]. Participants were instructed on hygienic collection of milk by qualified nursing staff. This included washing hands with soap and water before milk collection and using sterile containers and clean equipment for expression. Participants were asked to clean the nipple with soap and water before expressing milk. BM was expressed using electronic pumps, hand-operated pumps or manual expression (according to the donor’s usual method), and an aliquot removed with a sterile Pasteur pipette to a 15 mL plastic tube. Whole BM was stored at 4°C until collected by research staff, and transported to the research laboratory on ice. Between expression of BM and processing of the milk, samples were stored for a median of 8.29 hrs (range 1–50.9 hrs) in the refrigerator or in transport (on ice). The volume of milk donated to the study was recorded at each visit before processing. Samples were mixed gently and the cells were pelleted by centrifugation at 600 x *g* for 15 minutes at 4°C. The lipid layer and skim milk were removed with a Pasteur pipette, and the remaining cell pellet was washed twice in phosphate buffered saline (Gibco), and resuspended in 0.1–2.0 mL of flow cytometry buffer, described in the cell staining method below, to approximately one million cells/mL, for optimal cell to antibody ratios during staining and quantification of cells. The volumes of flow cytometry buffer that were used to resuspend cells and the original volume of milk processed were recorded for each sample so that the original cell concentration of the milk samples could be determined. Peripheral venous blood was collected from a single healthy adult donor into a sodium heparin Vacutainer (BD), and was mixed and stored at room temperature until staining.

### Determination of cell viability using visual counting methods

All cells enumerated by microscopy were analysed by a single operator using a Neubauer improved haemocytometer (BOECO, Germany). For each sample, two different aliquots of BM cell suspensions were stained by dilution of cell suspensions to approximately 1 x 10^5^–2.5 x 10^6^ cells/mL in a 0.4% Trypan blue solution (Sigma-Aldrich, Castle Hill, Australia) in PBS, or white cell counting fluid (2% glacial acetic acid containing crystal violet). Ten microliters of each suspension was pipetted into the counting chamber under the cover slip, and the average of three counts taken to calculate the concentration of cells, based on the volume in the chamber. Trypan blue stained cell counts were used to determine the non-viable cell concentration. A total cell concentration estimate was calculated by counting nucleated cells in white cell counting fluid. The proportion of non-viable cells was calculated based on the numbers of Trypan blue stained (non-viable) compared to total cells.

### Cell staining

Antibodies raised against human leukocyte antigens, stabilising fixative, FACSLyse, compensation beads and Trucount tubes were purchased from BD (North Ryde, Australia). Flow cytometry buffer was prepared in phosphate buffered saline (Gibco) supplemented with heat-inactivated fetal bovine serum (FBS; Sigma, Australia) at 2% v/v, 2% w/v bovine serum albumin (Sigma-Aldrich), and 0.01% w/v sodium azide (Sigma-Aldrich).

Fifty microliters of either BM cells suspended in flow cytometry buffer at approximately 1 x 10^6^ cells/mL or whole blood was incubated for 15 minutes at room temperature with a cocktail of six specific anti-human monoclonal antibodies (mAb): Phycoerythrin (PE) conjugated CD36 (clone CB38, catalogue #555455; 10 μL per sample), Allophycocyanin (APC) conjugated CD2 (RPA-2.10, 560642; 0.5 μL per sample), Alexa Fluor 647 conjugated CD294 (BM16, 561797; 0.5 μL per sample), APC-H7 conjugated CD16 (3G8, 560195; 2.5 μL per sample), V450 conjugated CD19 (HIB19, 560353; 0.25 μL per sample), and V500 conjugated CD45 (HI30, 560779; 1.25 μL per sample). The final dilution of antibody used was determined through titration experiments. Stained blood was treated with FACSLyse according to manufacturer’s specifications prior to further analysis to remove erythrocytes. After staining, BM and blood cells were washed once with 1 mL of flow cytometry buffer by centrifugation at 290 x *g* at 10°C for 3 min, then resuspended in 300 μL of BD stabilizing fixative and transferred to a Trucount tube. Fixed samples were stored at 4°C protected from light until analysis by flow cytometry, which for most samples was performed within 2 hrs, though the maximum allowed storage time for fixed cells before cytometry was 24 hrs based on previous experiments in our laboratory, which demonstrated the preservation of the staining characteristics under these conditions (data not shown).

### Flow cytometry

Flow cytometry was performed on a FACSCanto II (BD Biosciences) using FACSDiva software (BD Biosciences). Before sample analysis, the flow cytometer settings were checked using Cytometer Setup and Tracking beads (CS&T beads, BD) according to the manufacturer’s instructions. Compensation beads were used with single stains of each antibody in order to determine the compensation settings, and these were applied in FlowJo software (version 10.0.6, Tree Star, Ashland, OR, USA) after data collection. The same compensation matrix was applied to all samples. A side scatter (SSC) threshold level was set at 4,000 units to eliminate debris. Gain settings were optimised for detection of stained populations using Ultra Rainbow Calibration Particles (SpheroTech Inc., Lake Forest IL, USA), and kept consistent throughout the study.

Recorded data were compensated post-hoc and analysed using FlowJo software. Statistical tests were performed in SPSS (IBM) and Prism for Mac (version 5, GraphPad, La Jolla CA, USA). Specific antibody staining data were visualised using bi-exponential transformation in FlowJo software. Gates used to discriminate positive and negative staining cells in FlowJo were set according to fluorescence minus one (FMO) tests of milk and blood samples, and these gates were applied consistently to all samples, allowing for minor adjustments for SSC variability.

#### Gating strategy

Positive gated populations were selected using gates set through FMO tests, in which <1.7% of cells stained falsely positive for any antibody. Gate specificity selected using this method was confirmed by using the gating strategy on a single adult peripheral blood sample as a biological comparison control [21]. Prior to gating of leukocytes, Trucount beads were gated separately from cells in a SSC vs. PerCPCy5.5 visualisation; doublets were discriminated using a forward scatter (FSC)-H vs. FSC-A plot, and small particles excluded in a FSC vs. SSC plot (S1 Fig). Leukocytes in human milk were identified based on the method of Faucher *et al*., using orientating and specific gates [13] as described below.

**Fig 1:**
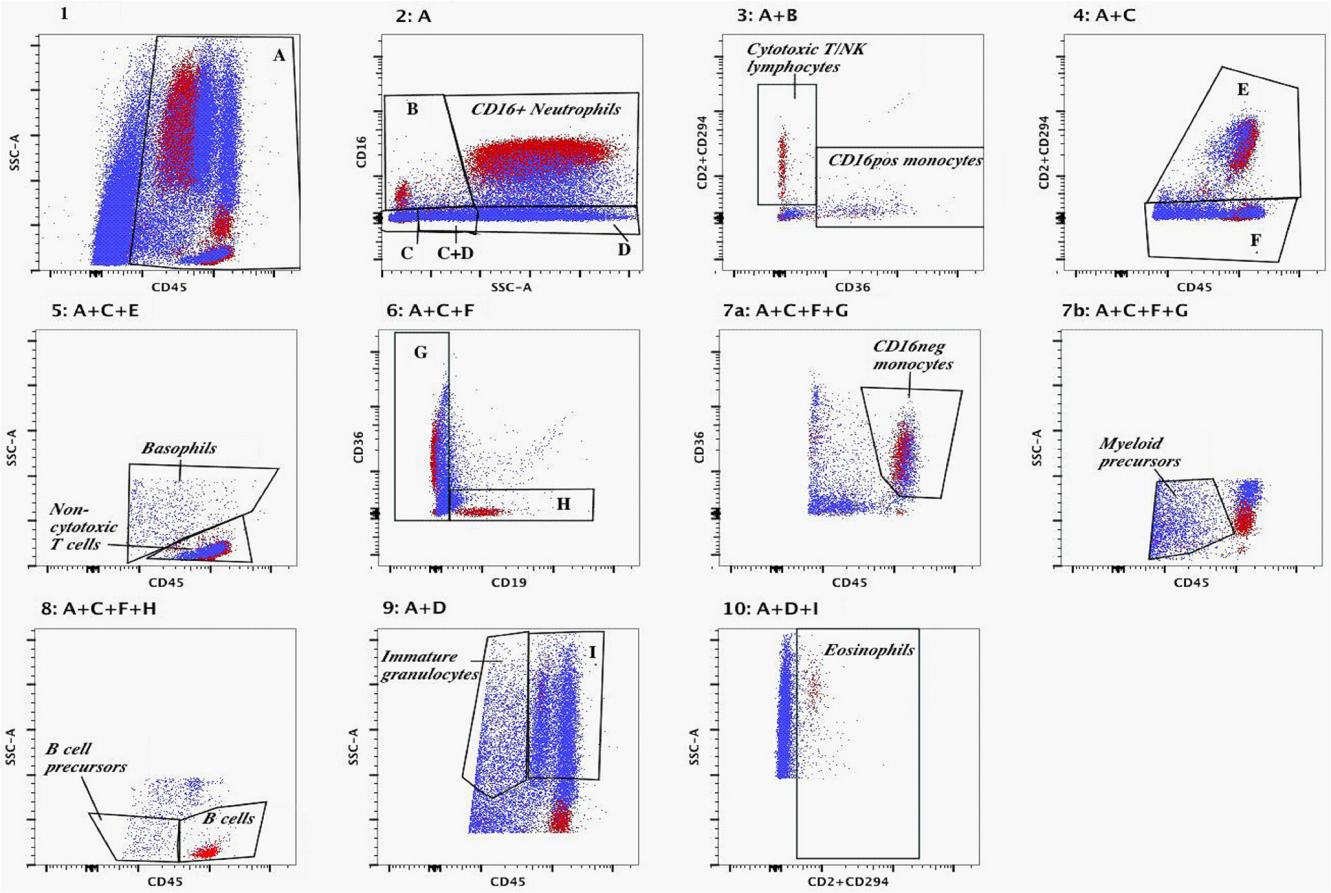
Overlay of gating strategy applied to peripheral adult blood (red) and to breast milk (blue), based on the method of Faucher *et al*. The sequences of gates leading to each panel are shown above each panel. Panel 1: CD45 positive cells were gated as shown in A. Panel 2: CD45+ cells identified in Panel 1 were separated based on CD16 staining and side scatter properties, including a C16+/SSClow gate (B), and two overlapping gates of CD16- cells (C and D), and the CD16+ neutrophil population were identified. Panel 3: CD45+/CD16+/SSClow cells identified in Panel 2 gate B were separated into cytotoxic T and NK lymphocytes and CD16+ monocytes based on CD2/CD294 and CD36 staining properties. Panel 4: CD45+/CD16-/SSClow–intermediate cells (gate C) were separated based on CD2/CD294 positive (gate E) or negative (gate F) populations. Panel 5: From Panel 4, CD2 and/or CD294 positive cells (gate E) were gated into non-cytotoxic T cells or basophils using side scatter and CD45 staining properties. Panel 6: From Panel 4, CD2/CD294- cells (gate F) were gated into CD19+/CD36- cells (gate H) or CD19- cells (gate G). Panel 7a: Cells gated in G in Panel 6 with CD45high and CD36+ were identified as CD16 negative monocytes. Panel 7b: From Panel 6 gate G, CD45low cells with low side scatter were identified as myeloid precursor cells. Panel 8: From Panel 6, CD19 positive cells (gate H) were discriminated into B cells or B cell precursors based on CD45 staining though both populations displayed low side scatter properties. Panel 9: From Panel 2, CD16- cells with intermediate to high side scatter (gate D) and CD45low staining were identified as immature granulocytes, and those with intermediate to high CD45 staining properties and high side scatter were separated into gate I. Panel 10: From Panel 9, cells in gate I were identified by positive CD2/CD294 staining as eosinophils.

The first orientating gate was selected using a SSC vs. CD45 plot where the cut-off for CD45+ cells was set using FMO control. Subsequently, a SSC vs. CD16 plot of CD45+ cells separated events into a specific SSChigh/CD16+ neutrophil gate, and three orientating gates (SSClow/CD16+, SSClow–int/CD16-, or SSChigh/CD16-). Subsequent orientating and specific gates for other populations are shown in Fig 1. CD16+ and CD16- monocytes, B lymphocytes, cytotoxic T and NK lymphocytes, non-cytotoxic T lymphocytes, neutrophils, eosinophils, basophils, immature granulocytes, myeloid precursors and B cell precursors were identified in colostrum, transitional and mature milk using this gating strategy.

#### CD45+ cell quantification using flow cytometry and Trucount beads

The absolute concentration of leukocytes in BM was calculated from the number of gated Trucount beads, as described by the manufacturer. Briefly, the number of gated CD45+ and Trucount bead events were combined with the data recorded on the volume of milk that the cell pellet was derived from to determine the concentration of cells in the original milk sample, using the formula;

Cell concentration (cells/mL) = (number of gated cells/number of gated Trucount beads) x (number of Trucount beads per test/total tested cell suspension volume) x (volume of flow cytometry buffer used to resuspend cell pellet /original milk volume recorded).

Leukocyte subset counts were determined in the same manner, and subset frequencies were determined by comparing specific subset concentrations to the total concentration of CD45+ cells in the sample.

### Analysis of the effects of maternal infection on breast milk composition

Collection of maternal clinical data including medications, health status and pregnancy information was performed using medical record review for each individual from hospital admission to discharge during birth, and at any subsequent hospital admissions during the first month postpartum. After hospital review, data on maternal health were collected through self-reported questionnaires covering the previous seven days at the time of milk donation. Maternal infection was considered present based on any evidence of chorioamnionitis (either with or without positive microbiological culture, where available tissues (n = 36) were examined by an experienced histopathologist according to the method of Redline *et al*. [22]), or a positive microbiological culture or symptoms of infection requiring treatment with antibiotics prescribed by a physician. Milk from individuals with known infection and without infection were compared at the three collection time points.

### Statistical analyses

Categorical data were summarised using frequency distributions, and continuous data was summarized using means and standard deviations or median and interquartile ranges, as appropriate. Nonparametric tests were used for comparisons of continuous outcomes, due to lack of normality evaluated using Shapiro-Wilk normality tests. Differences in outcomes between the four GA groups were compared using Kruskal-Wallis tests with Dunn’s multiple comparison post-test. Concentrations of cells in the same mother in colostrum, transitional or mature milk were examined using a Freidman test and individual sampling periods were compared using Dunn’s multiple comparison post-test. Milk concentration comparisons with and without infection were compared with Mann-Whitney tests. Comparisons were made between individuals for whom data on health status was available at the time of milk collection only. Spearman correlation (denoted ‘ρ’) was used to measure the association between GA at birth and factors in milk.

Statistical analysis was performed using SPSS statistical analysis software (version 20, IBM, Armonk, NY, USA). p-values <0.05 were considered statistically significant.

## Results

### Participant characteristics

The clinical characteristics of the sixty study participants are shown in Table 1. The number of samples analysed in each GA group are shown in Table 2.

**Table 1:** Clinical characteristics of the sixty study participants. Bolded p-values indicate those that were considered statistically significant. Continuous variables gestation period and maternal age were compared between groups using one-way analysis of variance, and categorical variables compared using Fisher exact Chi-squared tests. EP = extremely preterm, VP = very preterm, MP = moderately preterm, SD = standard deviation.

**Clinical characteristic**	Value	EP (n = 15)	VP (n = 15)	MP (n = 15)	Term (n = 15)	p-value
Gestation period (mean ±SD)	Weeks	26.1±1.2	30.2±1.3	34.4±1.3	39.4±0.77	**<0.001**
Maternal age (mean±SD)	Years	31.3±5.2	30.1±6.9	31.6±6.9	29.7±6.0	0.879
Maternal Infection (n, % providing milk)	Colostrum	3 (30)	6 (55)	3 (27)	1 (11)	**0.016**
	Transitional milk	2 (20)	3 (23)	0 (0)	3 (25)	0.085
	Mature milk	2 (20)	2 (17)	1 (11)	2 (13)	0.826
Mode of delivery (n, %)	Vaginal	9	5	8	9	0.448
	Caesarean section	6	10	7	6	

**Table 2:** Total leukocyte and subset concentrations in breast milk measured across the first month of lactation. Median [interquartile range] values of total leukocytes and leukocyte subsets per millilitre of breast milk detected using flow cytometry in preterm and term mother groups in colostrum, transitional milk and mature milk. Symbol ^a^ with bolded text denotes a significantly different comparison in Kruskal-Wallis tests comparing gestational age groups after adjusting for multiple comparisons. Other comparisons were not statistically significant. EP = extremely preterm, VP = very preterm, MP = moderately preterm.

	Colostrum				Transitional milk				Mature milk			
	EP (n = 10)	VP (n = 11)	MP (n = 11)	Term (n = 9)	EP (n = 10)	VP (n = 13)	MP (n = 12)	Term (n = 12)	EP (n = 10)	VP (n = 12)	MP (n = 9)	Term (n = 15)
CD45 cells/mL	271,000 [95,200–439,000]	96,000 [73,700–352,000]	129,000 [67,700–360,000]	184,000 [111,000–291,000]	69,550 [20,300–136,000]	26,000 [14,400–54,200]	27,250 [10,450–63,950]	32,450 [10,450–66,150]	27,250 [14,400–48,700]	12,500 [7,360–23,700]	44,700 [28,200–77,400]	14,700 [7,420–56,500]
CD16+ monocytes	2,415 [1,420–14,100]	3,850 [1,930–7,920]	2,490 [1,070–4,890]	3,060 [903–5,800]	657 [370–1,450]	486 [310–1,060]	346 [248–646]	364 [170–772]	706 [203–1,130]	225 [123–435]	686 [384–1,110]	307 [164–1,160]
Cytotoxic T&NK cells	2,020 [389–6,010]	1,530 [208–4,510]	1,020 [722–1,940]	945 [468–1,250]	198 [86–701]	135 [79–835]	288 [145–648]	222 [66–756]	452 [81–1,220]	**109 [59–218]** ^**a**^	**430 [198–947]** ^**a**^	340 [128–513]
Basophils	7,125 [3,460–10,600]	2,780 [1,330–12,400]	4,490 [2,440–9,050]	2,460 [1,490–3,410]	654 [174–1,240]	611 [158–851]	591 [345–982]	435 [172–1,320]	235 [106–369]	263 [98–374]	1,550 [340–2,320]	370 [96–571]
Non-cytotoxic T cells	29,045 [8,760–62,500]	14,800 [4,380–26,500]	24,800 [2,860–41,200]	5,410 [4,640–16,300]	2,130 [1,480–5,750]	1,860 [732–6,940]	3,545 [335–4,210]	1,740 [524–4,300]	1,205 [400–5,330]	647 [397–1,695]	4,360 [953–9,530]	1,290 [250–4,040]
CD16- monocytes	2,805 [1,480–7,850]	2,350 [997–4,720]	2,460 [1,290–14,500]	1,990 [233–5,800]	198 [46–971]	183 [71–776]	357 [252–1,128]	531 [89–801]	106 [21–916]	146 [43–344]	1,070 [170–2,370]	213 [28–501]
Myeloid precursors	24,100 [13,700–63,200]	15,500 [6,690–84,300]	19,600 [10,100–80,300]	16,900 [14,200–21,300]	7,475 [4,040–57,900]	4,050 [1,930–8,930]	5,210 [1,295–8,095]	6,295 [2,480–11,750]	1,173 [532–4,460]	1,230 [520–2,790]	4,060 [2,550–6,430]	1,490 [559–4,560]
B cell precursors	2,395 [1,720–12,700]	3,100 [1,970–10,300]	1,800 [689–5,110]	7,720 [1,620–8,370]	**1,825 [1,360–2,840]** ^**a**^	530 [305–917]	**233 [158–555]** ^**a**^	485 [207–1,235]	298 [132–1,130]	81 [47–258]	124 [89–194]	135 [74–285]
B cells	1,311 [356–8,060]	716 [306–2,860]	470 [227–868]	982 [333–1,230]	414 [63–552]	167 [57–260]	82 [41–118]	149 [73–230]	**177 [46–313]** ^**a**^	**24 [14–89]** ^**a**^	160 [57–221]	59 [26–127]
Neutrophils	25,450 [9,520–74,500]	15,700 [6,410–26,700]	20,600 [7,840–25,500]	23,500 [1,980–65,900]	6,785 [1,640–15,400]	2,250 [1,810–4,740]	4,095 [1,635–12,250]	5,640 [1,221–14,425]	7,665 [2,950–13,400]	3,745 [2,280–5,765]	9,310 [7,610–10,500]	2,560 [1,490–25,700]
Eosinophils	4,040 [1,550–8,010]	2,650 [1,160–10,200]	3,690 [2,270–5,520]	1,760 [1,040–2,890]	397 [78–915]	228 [132–933]	408 [187–859]	371 [75–1,685]	157 [31–402]	164 [51–461]	976 [181–2,620]	269 [110–395]
Immature granulocytes	19,400 [13,100–35,800]	7,780 [4,300–20,600]	10,100 [2,580–35,000]	12,600 [8,410–26,500]	7,850 [1,660–35,900]	3,280 [1,300–6,870]	2,970 [1,615–5,435]	2,945 [2,160–12,230]	4,390 [949–16,100]	2,510 [1,295–8,595]	9,100 [7,600–11,000]	2,050 [775–14,600]

### Milk cell counts and viability

The median number of objects with cell-like appearance in all samples using white cell counting fluid and microscopy was 313,500 per millilitre (range 34,300–8,020,000 per millilitre). The time from sample collection to processing was short for most samples (median 8.29 hrs, range 1–50.9 hrs) and the corresponding percentage of dead cells was low (median 1.46% dead, range 0–11.52%, respectively). The percentage of dead cells identified by Trypan blue exclusion correlated with the period between BM collection and processing (ρ = 0.43, p<0.001), but did not correlate with CD45+ cell concentrations or the proportion of leukocytes that were identifiable. There were no significant differences in cellular viability or storage times between the preterm and term mother milk samples at any time point.

**Fig 2:**
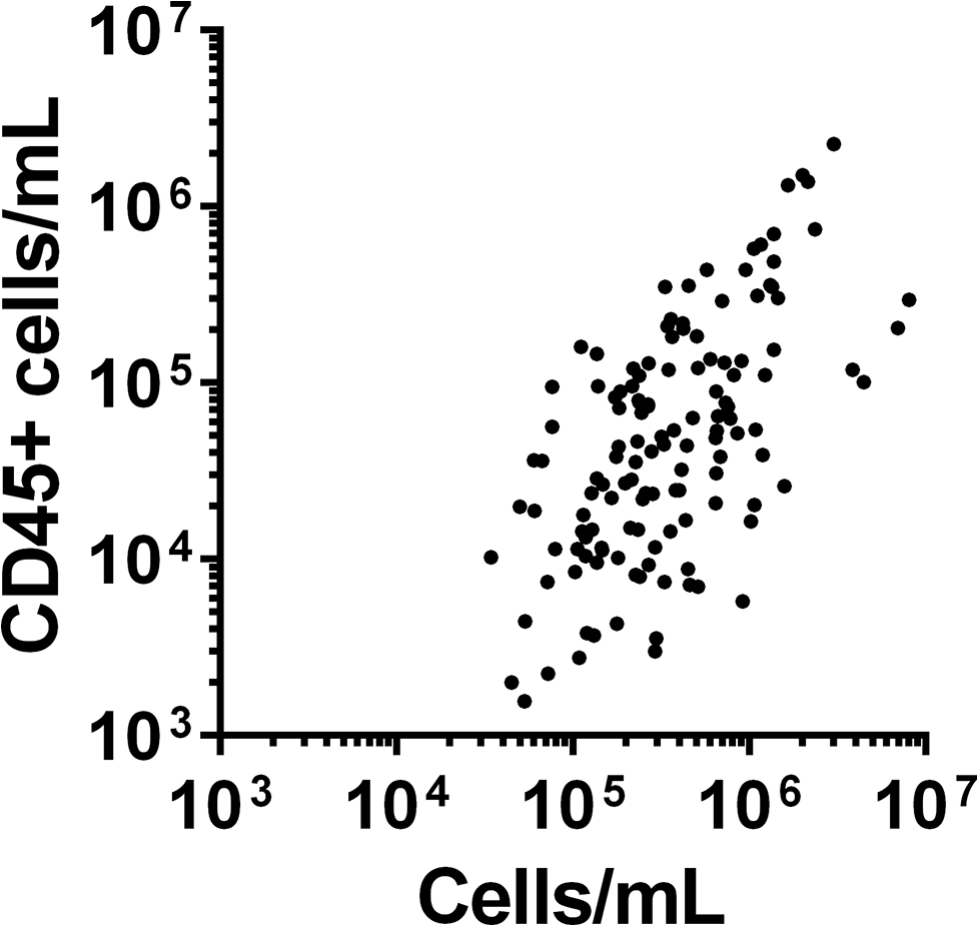
Comparison of the total cell concentrations obtained from milk samples with different methods. Figure shows scatterplot of total cell counts obtained through microscopy on x-axis, compared with total CD45+ leukocyte concentrations obtained with flow cytometry on the y-axis. All data are shown on a log scale.

In comparison to the concentration of CD45+ cells obtained with flow cytometry using Trucount beads, the total nucleated cell counts obtained with microscopy were consistently higher, but a significant correlation between the two measures was observed (ρ = 0.6, p<0.001; Fig 2).

### Gating strategy

The application of the whole blood gating strategy for assessing leukocytes in BM samples in mothers is shown in Fig 1, comparing a representative BM sample to an adult blood sample. In colostrum, a median 64% (range 37%–85%) of leukocytes were members of identifiable leukocyte subsets based on the Faucher blood phenotype gating strategy, increasing to 77% (range 37%–95%) in transitional milk and 82% (range 65%–98%) in mature milk (Friedman test p<0.001). In BM, there were additional CD45+ populations that could not be categorised according to the Faucher gating method. These ungated leukocyte populations can be seen outside the established gates in Panels 3, 6, 8, and 10.

### Changes in CD45+ cell concentration through the stages of lactation

The distributions of BM concentrations of total CD45+ cells in all GA groups were significantly different at the different collection periods (p<0.001). The concentration of leukocytes in colostrum (median 146,000 cells/mL, range 8,470–1,510,000) was significantly higher than in both transitional milk (median 27,500 cells/mL, range 1,570–2,260,000; p<0.001) and mature milk (median 23,650, range 2,000–577,000; p<0.001). No significant difference between the transitional milk and mature milk leukocyte concentrations was observed.

**Fig 3:**
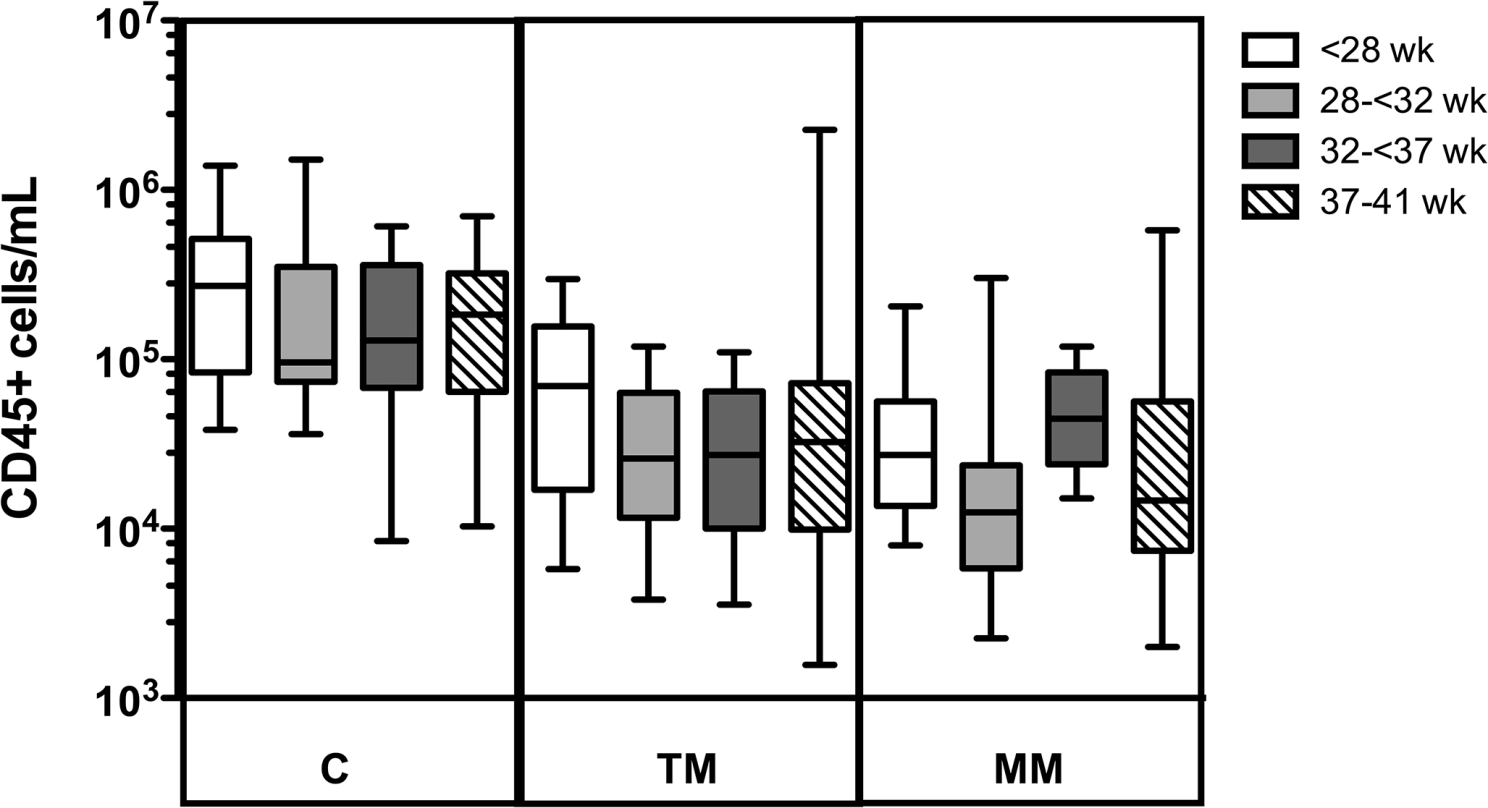
Leukocyte concentrations in breast milk samples. Boxplots showing the total concentration of leukocytes (cells/mL) on a log scale in colostrum (C, n = 41), transitional milk (TM, n = 47), and mature milk (MM, n = 46), in extremely preterm (white), very preterm (light grey), moderately preterm (dark grey) and term (striped) donors.

There were no significant differences in total leukocyte concentration between GA groups in colostrum, transitional milk or mature milk (Fig 3). The concentration of leukocytes was negatively correlated with the volume of BM expressed (ρ = -0.317; p<0.01), and therefore, differential analyses of the frequency of leukocyte subsets, in addition to total leukocyte concentrations, were performed.

### Changes in breast milk leukocyte composition from colostrum to mature milk

**Fig 4:**
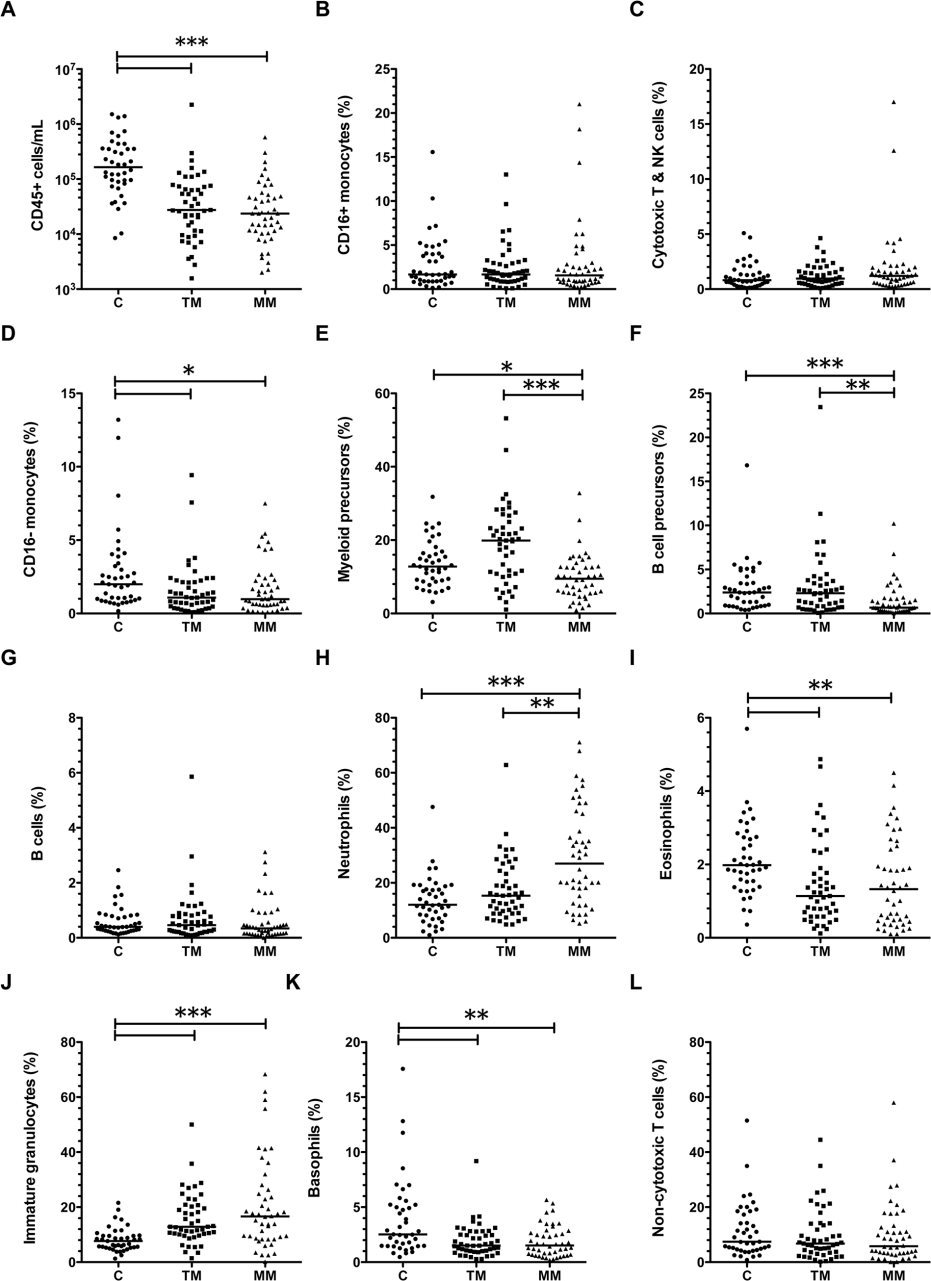
Changes to the composition of breast milk through the first month of lactation. Line shows the median value for that milk sampling time point. Data show A) concentrations of total CD45+ cells in colostrum (C), transitional milk (TM) or mature milk (MM); B-L) frequencies of leukocyte subsets in colostrum (C), transitional milk (TM) and mature milk (MM) from all donors. *p<0.05 in post-test comparing different stages of lactation; for each comparison, number of symbols indicates p-value (*p<0.05, **p<0.01, ***p<0.001).

The median frequencies of leukocyte subsets identified in colostrum, transitional milk, and mature milk are shown in Fig 4. A proportion of leukocytes did not fall into set gates for blood leukocytes (median at each time point 18–36% across GA groups). The percentage of leukocytes that were identified as blood subsets in milk was positively correlated with the number of days postpartum that the milk was expressed (ρ = 0.604, p<0.001). Of the identified cells, the major leukocytes present were myeloid precursors (median 9–20%), neutrophils (median 12–27%), immature granulocytes (median 8–17%), and non-cytotoxic T cells (median 6–7%). The relative median frequencies of neutrophils and immature granulocytes of total leukocytes significantly increased from colostrum to mature milk (Fig 4H and 4J), whereas the relative frequencies of CD16- monocytes, myeloid precursors, B cell precursors, eosinophils, and basophils decreased over the first month postpartum (Fig 4 parts D, E, F, I and K; p<0.05).

### Effects of gestation on milk leukocyte concentration and frequencies

Some minor differences in GA groups were observed in the concentration of specific leukocyte subsets at different sampling points. In colostrum, the relative frequencies of non-cytotoxic T cells and B lymphocytes in the total leukocyte populations were negatively correlated with increasing gestation (ρ = -0.35, p = 0.03 and ρ = -0.319, p = 0.04, respectively).

In transitional milk, the concentration of B cell precursors was significantly higher in extremely preterm mothers compared to moderately preterm mothers (median 1,825 cells/mL vs. 233 cells/mL, p = 0.02; Table 2), and the frequency of CD16 negative monocytes was significantly lower in extremely preterm mothers compared to moderately preterm mothers (median 0.34% vs. 2.13%, p = 0.03; Table A in S1 File). In transitional milk, the frequency of neutrophils was positively correlated with gestation (ρ = 0.305; p = 0.04).

In mature milk, total cytotoxic T and NK cells concentrations were significantly lower in very preterm compared to moderately preterm mothers (median 109 vs. 430 cells/mL; p = 0.04). B lymphocyte concentration was significantly higher in extremely preterm compared to very preterm mothers (median 177 vs. 24 cells/mL; p = 0.04). In mature milk, no significant correlations between total leukocyte or subset frequencies and gestation were observed.

### Effects of bacterial infections on leukocyte concentration and frequencies

The total number of CD45+ cells did not significantly differ in BM of mothers with or without recent infection (for more information, refer to Table B in S1 File). There were no differences in milk leukocyte concentrations or leukocyte subset frequencies between those with or without infection in colostrum. However, in transitional milk, those with reported bacterial infections around the time of the donation (n = 7) had significantly lower frequencies of basophils than those who were well (n = 40) (median 0.91% vs. 1.68%; p = 0.02). Furthermore, bacterial infections (n = 6 vs. not infected n = 40) at the time of mature milk sampling were also associated with lower frequencies of basophils (median 0.72% vs. 1.67%; p = 0.03).

## Discussion

This study aimed to characterise leukocytes (CD45+) in human preterm and term BM during the first month of life using a flow cytometry-based, extended differential leukocyte counting method. We successfully identified leukocytes in BM at all stages of lactation examined using the leukocyte gating panel described by Faucher *et al*. [13]. The majority of CD45+ cells in BM showed a similar phenotype to blood cells, though differences in the frequencies of leukocyte subsets and other non-circulating cell populations were observed compared to blood. There were no differences in total leukocyte concentrations between the GA groups, whereas some differences were found in specific leukocyte subset concentrations and frequencies between GA groups. As lactation progressed, the concentration of total leukocytes in BM decreased. We did not observe significant changes in the concentration of total leukocytes in BM of mothers with clinical infections, though minor alterations to leukocyte subset frequencies were observed during maternal infections.

Traditionally, BM leukocytes have been identified using blood smear stains and cytology, and leukocyte populations were reported to be composed mainly of macrophages, neutrophils, T lymphocytes, B lymphocytes, and monocytes [23]. However, visual identification can result in misidentification and overestimation of BM leukocyte concentration, whereas multicolour flow cytometry provides superior identification and quantification of leukocytes [23]. We found a clear and consistent relationship in cell concentrations when directly comparing both methods, though non-leukocyte cells are clearly also present in BM.

Despite the benefits of flow cytometry, there are limitations. The use of appropriate controls and cytometer settings are critical to reduce the likelihood of misidentification of cells [21, 24, 25]. We selected a biological control (blood) to set the cut-off for positive CD45 staining and this is the major source of potential error, since this is the only gate with a single criterion for cell selection. We did not perform FMO controls for each milk sample, instead an indication of the background staining was taken from three milk and one blood control tested (<1.7% in all cases), though the proportion of background staining could have been greater in individual milk samples. Reassuringly, the majority of cells that we identified as CD45+ were subsequently gated into specific leukocyte populations. In future studies, additional biological controls for gating positive populations may be used in combination with sample-specific controls.

Using a multicolour flow cytometry panel validated to identify CD45+ leukocyte subsets in human blood [13], we were able to identify and quantify equivalent leukocytes in BM. Recently, researchers have applied single colour and multicolour flow cytometry to human milk to identify a broader range of cells than previously appreciated, including B lymphocytes and T lymphocyte subsets in term colostrum, in addition to NK and NK-T cells [26, 27]. Though the functionality of myeloid-derived cells in milk suggests that these cells may be more differentiated than in blood [28], it is not known with the exception of B lymphocytes [29], whether the morphologies and frequencies of specific BM leukocyte subsets, only identifiable by flow cytometry, correspond to those found in blood. We found evidence of additional leukocytes not previously recognised in milk, including immature granulocytes and myeloid precursors. Further CD45+ cell subsets were not readily identifiable, but could include macrophages, plasma cells, dendritic cells and others. Additional cell markers such as CD14, CD64, HLA-DR, CD86 and CD11c for macrophage and dendritic cells [30], CD138 for plasma cells [31] and selection of combinations of other markers of haematopoietic progenitor cells such as CD34, CD38, CD135 and CD10 could be used for granulomonocytic and other precursor populations [32], but will need to be validated for flow cytometry in future BM research, particularly for low or negative CD45-staining cells, where immature leukocytes and rare leukocyte types in blood have potential to be misidentified [33].

We found a clear inverse relationship between maturational stage of the BM and the concentration of leukocytes. Despite temporal changes to cell numbers, substantial (10^3^–10^6^ cells/mL) leukocyte numbers were universally present at all sampling time points, with inter-individual variability. Recent studies have reported a significant association between increased numbers of leukocytes in BM and infections of both the mother and the infant [27, 34]. The high number of leukocytes found in the current study across all GA groups may relate to a current illness of either the mother or her infant that was not identified and/or reported, and therefore was not accounted for, or it could also be related to methodological differences between studies in how leukocytes were identified. Importantly, we noted much lower frequencies of neutrophils and monocytes than previously reported (median 12.4% neutrophils and 3.7% monocytes in this study, compared to 28–48.8% and 40.8–61% of colostrum leukocytes, respectively) [26, 35]. This is possibly due to similarities in visual appearance of other cell types, indistinguishable by microscopy. The employment of flow cytometry-based methods combined with bead count methods in a single platform may significantly expand the ability to describe and improve the accuracy of reporting of BM leukocyte data in the future. One limitation of this work was the necessity to collect BM that had been refrigerated prior to processing, which resulted in inclusion of low numbers of non-viable cells. However, this was done to avoid further burden on the donors, particularly mothers of extremely preterm infants. Future work could include a viability marker to directly assess for non-viable cells. In addition, we cannot account for any potential effects that different methods of breast milk expression might have had on the measurements made in the milk, as data were not collected about the method used for each sample.

It has previously been reported that moderately preterm mothers’ BM contains higher concentrations of leukocytes compared to term mothers [12, 36]. In contrast, Goldman *et al*. reported that the concentrations of leukocytes were lower in preterm BM [37], whereas Rodriguez *et al*. found leukocyte numbers were not different between preterm and term mothers [38]. We did not observe significant effects of preterm birth on concentration of leukocytes at any stage of lactation, but we did find a negative correlation between the frequency of B lymphocytes and non-cytotoxic T lymphocytes with gestation in colostrum. We also found a positive correlation between the frequency of neutrophils and gestation in transitional milk. These findings may reflect altered immune activation status or increased expression of proliferation or chemotactic factors for certain cell types in the mammary gland of mothers of preterm infants, and merit further investigation. Previous studies did not include mothers of extremely preterm infants or use specific staining methods to quantify total leukocytes; therefore, the possibility of comparison to this work is limited, particularly considering that our method does not identify macrophages. Our sampling period for colostrum was typically on day 4 postnatally, and we may have missed the changes observed in other studies between days 1–3.

Research by other groups suggests that the functionality of preterm colostrum leukocytes (e.g. bacterial phagocytosis and killing as well as cellular proliferation) appears similar to term BM [38, 39]. Therefore, this study suggests that the increased risk of infection in preterm infants is not associated with deficiencies in leukocyte concentration or activity in fresh unprocessed preterm BM, nor deficiencies in milk antimicrobial proteins and peptides [40]. However, preterm infants often receive low volumes of human BM (typically refrigerated) or they are given pasteurised donor human milk (PDHM) with inactivated leukocytes [41]. BM-derived leukocytes may aid in protecting infants from infection through normal protective functions such as phagocytosis and production of antimicrobial peptides, and have been found in the peripheral circulation and distant tissues of animal models after ingestion [10, 42], with observed effects on blood leukocyte populations in both animal and human studies [43–46]. However, it is unlikely that low consumption of leukocytes, independent of the many other protective functions of the soluble molecules in milk, is responsible for the increased infection risk in preterm infants fed PDHM and those consuming low BM volumes [1, 47].

Contrary to previous reports of increased leukocyte concentrations in BM in maternal or infant infections [27, 34], we did not observe significant increases in absolute leukocyte concentration during infections in our study. However, sensitivity for detecting differences was limited (below 2-fold in colostrum and below 3-fold in other samples) by the small number of infected mothers. Moreover, the presence of non-reported infections in this population cannot be excluded. Significantly, the majority of donors were taking at least one medication, such as antibiotics, analgesics or anti-inflammatory drugs at one point during the study. This study was not powered to assess possible effects of medications on leukocytes. Overall, our data suggest that cellular composition of BM is affected by stage of lactation.

This is the first study to comprehensively identify and characterise immune cell subsets in preterm BM by flow cytometry. Despite the technological advances of multicolour flow cytometry, the progress of characterisation of BM leukocytes has been relatively neglected compared to blood. The characterisation of the cellular concentrations and their function in BM is critical to understand how BM protects the infant and/or mammary gland from infections. Fresh preterm BM is not deficient in leukocytes and infants receiving lower doses of BM, frozen or pasteurised human BM may be disadvantaged. This work demonstrates that BM contains a greater variety and complexity of leukocyte subsets than previously appreciated.

## Supporting Information

S1 Fig Preliminary gates of the flow cytometry method. Gates were used to select and exclude Trucount beads (1), remove doublets (2), and exclude non-cellular material and bacteria (3), respectively.
(TIFF)

S1 File Table A in S1 File. Leukocyte subset frequencies in milk collection across the first month of lactation. Median [interquartile range] values of relative frequencies of leukocyte subsets detected using flow cytometry in preterm and term mother groups in colostrum, transitional milk and mature milk Symbol ^a^ with bolded text denotes significantly different comparison in Kruskal Wallis test comparing gestational age groups after adjusting for multiple comparisons. EP = extremely preterm, VP = very preterm, MP = moderately preterm. **Table B in S1 File. Prevalence of reported bacterial infections in milk donors during the sample collection period.** Abbreviations: URTI = upper respiratory tract infection; UTI = urinary tract infection. Infections around the time of colostrum were reported in medical records, after hospital discharge, infections were self-reported (most transitional and mature milk collections).
(DOCX)
