# Supplementary figures and images for: Leukocyte Populations in Human Preterm and Term Breast Milk Identified by Multicolour Flow Cytometry
Source: PLoS One. 2015 Aug 19;10(8):e0135580. doi: 10.1371/journal.pone.0135580 (PMC4545889; doi:10.1371/journal.pone.0135580)

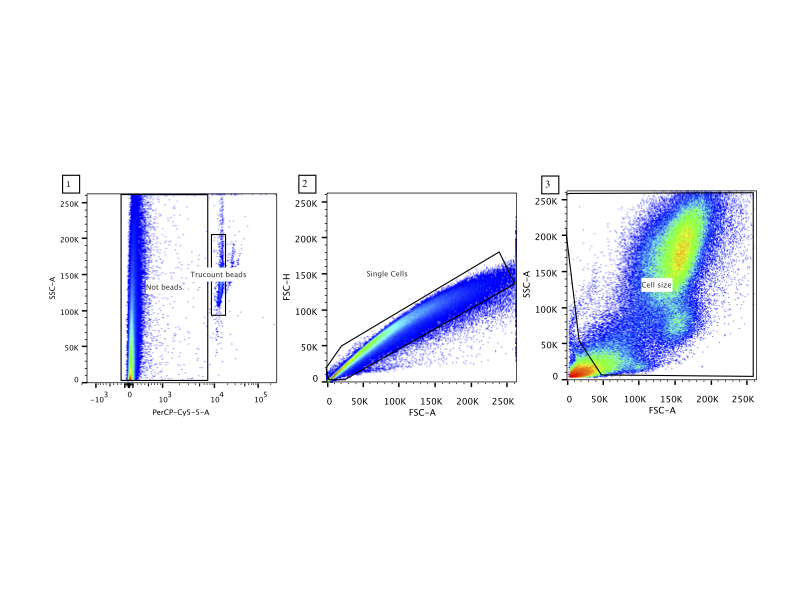

Supplement: S1 Fig — Gates were used to select and exclude Trucount beads (1), remove doublets (2), and exclude non-cellular material and bacteria (3), respectively. (TIFF) [file pone.0135580.s001.tiff]
