# Supplementary material for: Leukocyte Populations in Human Preterm and Term Breast Milk Identified by Multicolour Flow Cytometry
Source: PLoS One. 2015 Aug 19;10(8):e0135580. doi: 10.1371/journal.pone.0135580 (PMC4545889; doi:10.1371/journal.pone.0135580)
Supplement: S1 File — Median [interquartile range] values of relative frequencies of leukocyte subsets detected using flow cytometry in preterm and term mother groups in colostrum, transitional milk and mature milk Symbol a with bolded text denotes significantly different comparison in Kruskal Wallis test comparing gestational age groups after adjusting for multiple comparisons. EP = extremely preterm, VP = very preterm, MP = moderately preterm. Table B in S1 File. Prevalence of reported bacterial infections in milk donors during the sample collection period. Abbreviations: URTI = upper respiratory tract infection; UTI = urinary tract infection. Infections around the time of colostrum were reported in medical records, after hospital discharge, infections were self-reported (most transitional and mature milk collections). (DOCX) [file pone.0135580.s002.docx]

|  | Colostrum | | | | Transitional milk | | | | Mature milk | | | |
| --- | --- | --- | --- | --- | --- | --- | --- | --- | --- | --- | --- | --- |
|  | EP  (n=10) | VP  (n=11) | MP  (n=11) | Term  (n=9) | EP  (n=10) | VP  (n=13) | MP  (n=12) | Term  (n=12) | EP  (n=10) | VP  (n=12) | MP  (n=9) | Term  (n=15) |
| CD16+ monocytes | 1.42 [.87-1.89] | 1.70 [1.42-5.23] | 1.93 [0.88-3.67] | 2.00 [0.89-4.91] | 1.29 [0.49-3.28] | 1.79 [1.19-2.93] | 1.83 [1.02-3.03] | 1.86 [0.88-2.50] | 1.80 [0.42-7.89] | 1.43 [0.85-2.28] | 1.17 [0.93-2.39] | 2.06 [0.82-4.07] |
| Cytotoxic T&NK cells | 0.73 [0.28-1.28] | 0.84 [0.22-2.08] | 0.98 [0.68-1.50] | 0.64 [0.48-0.94] | 0.63 [0.10-1.49] | 0.87 [0.36-1.54] | 1.37 [0.61-2.10] | 0.94 [0.72-1.32] | 1.13 [0.34-2.12] | 0.89 [0.46-1.37] | 1.23 [0.56-2.12] | 1.45 [0.77-2.28] |
| Basophils | 2.85 [1.79-5.21] | 2.89 [1.49-5.22] | 4.43 [1.47-7.06] | 2.22 [1.26-2.52] | 1.18 [.53-1.64] | 1.93 [1.18-2.78] | 2.00 [1.11-3.28] | 1.36 [1.06-2.44] | 0.74 [0.55-1.66] | 1.52 [1.14-2.27] | 2.46 [1.21-2.65] | 1.77 [0.53-3.78] |
| Non-cytotoxic T cells | 10.12 [6.80-18.51] | 10.65 [4.42-18.54] | 11.47 [5.76-19.16] | 4.64 [3.67-5.17] | 2.54 [1.94-8.30] | 7.73 [5.09-14.12] | 9.22 [5.53-20.85] | 6.61 [4.03-8.93] | 4.78 [3.29-17.69] | 5.66 [3.53-12.14] | 9.79 [2.82-10.97] | 7.83 [1.34-12.63] |
| CD16- monocytes | 1.58 [0.87-3.42] | 2.45 [0.76-3.23] | 2.54 [1.90-4.11] | 1.16 [0.92-1.99] | **0.34 [0.22-0.73]^a^** | 1.35 [0.70-1.76] | **2.13 [0.95-2.42]^a^** | 1.02 [0.68-2.12] | 0.38 [0.15-0.97] | 1.49 [0.84-2.13] | 1.28 [0.64-2.64] | 0.89 [0.35-2.65] |
| Myeloid precursors | 13.60 [8.72-21.00] | 14.47 [9.58-19.64] | 13.18 [9.45-16.44] | 10.99 [6.95-13.13] | 21.65 [13.36-31.26] | 21.67 [11.37-24.96] | 14.55 [10.11-20.22] | 20.10 [8.39-25.80] | 5.71 [2.98-10.14] | 8.29 [5.89-13.23] | 10.13 [7.68-14.66] | 10.51 [6.06-12.60] |
| B cell precursors | 1.45 [0.83-3.44] | 2.93 [1.32-3.47] | 1.74 [0.72-2.66] | 2.87 [2.35-5.19] | 2.55 [1.44-3.78] | 1.93 [0.84-3.68] | 0.89 [0.46-3.07] | 2.62 [0.81-3.79] | 1.59 [0.31-3.19] | 0.66 [0.43-1.08] | 0.29 [0.20-0.38] | 1.00 [0.36-1.82] |
| B cells | 0.54 [0.29-0.89] | 0.44 [0.31-0.84] | 0.38 [0.22-0.50] | 0.31 [0.27-0.53] | 0.43 [0.25-1.64] | 0.61 [0.23-0.81] | 0.29 [0.17-0.51] | 0.47 [0.20-0.83] | 0.41 [0.18-0.91] | 0.22 [0.14-0.42] | 0.19 [0.14-0.49] | 0.44 [0.13-1.04] |
| Neutrophils | 11.04 [5.97-19.15] | 12.02 [4.50-17.63] | 11.58 [8.16-17.35] | 16.09 [10.23-18.73] | 10.26 [6.08-16.41] | 10.15 [8.83-15.33] | 17.47 [11.98-28.16] | 18.43 [15.09-26.48] | 19.57 [9.42-48.93] | 31.93 [20.02-47.53] | 22.20 [18.25-33.05] | 30.37 [11.68-45.09] |
| Eosinophils | 1.66 [1.28-2.85] | 2.19 [1.99-2.91] | 2.17 [1.75-3.51] | 1.83 [1.38-1.92] | 0.64 [0.25-1.11] | 1.41 [1.10-2.41] | 1.36 [0.80-2.35] | 0.96 [0.58-2.36] | 0.53 [0.30-1.37] | 1.36 [0.63-2.17] | 1.95 [0.91-3.38] | 1.45 [0.44-2.64] |
| Immature granulocytes | 7.41 [5.62-10.57] | 7.68 [5.62-9.56] | 8.34 [4.64-9.72] | 6.26 [5.74-9.58] | 17.96 [10.70-27.61] | 14.97 [9.69-20.88] | 11.49 [9.36-16.54] | 11.89 [10.37-18.04] | 13.48 [3.44-36.10] | 21.68 [9.33-39.71] | 16.73 [13.88-18.21] | 13.89 [7.60-19.27] |

**Supplementary Table A. Leukocyte subset frequencies in milk collection across the first month of lactation.** Median [interquartile range] values of relative frequencies of leukocyte subsets detected using flow cytometry in preterm and term mother groups in colostrum, transitional milk and mature milk. Symbol ^a^ with bolded text denotes a significantly different comparison in Kruskal Wallis test comparing gestational age groups after adjusting for multiple comparisons. EP=extremely preterm, VP=very preterm, MP=moderately preterm.

|  | Chorioamnionitis | | UTI | Skin | Gynaecological | Mastitis | Gastroenteritis | URTI | Unspecified | Total |
| --- | --- | --- | --- | --- | --- | --- | --- | --- | --- | --- |
|  | No maternal sepsis | With Sepsis |  |  |  |  |  |  |  |  |
| Colostrum | 13 | 1 | 1 | 1 | 0 | 0 | 0 | 0 | 0 | 16 |
| Transitional milk | 0 | 0 | 2 | 1 | 3 | 1 | 0 | 0 | 0 | 7 |
| Mature milk | 0 | 0 | 1 | 1 | 0 | 1 | 1 | 1 | 1 | 6 |

**Supplementary Table B. Prevalence of reported bacterial infections in milk donors during the sample collection period.** Abbreviations: URTI=upper respiratory tract infection; UTI=urinary tract infection. Infections around the time of colostrum were reported in medical records, after hospital discharge, infections were self-reported (most transitional and mature milk collections).
